# Glycogen Synthase Kinase-3beta regulates Snail and beta-catenin during gastrin-induced migration of gastric cancer cells

**DOI:** 10.1186/1750-2187-5-9

**Published:** 2010-07-16

**Authors:** Prajna Mishra, Subramanian Senthivinayagam, Ajay Rana, Basabi Rana

**Affiliations:** 1 Department of Medicine, Division of Gastroenterology, Hepatology and Nutrition, Loyola University Chicago, 2160 South First Avenue, Maywood, IL 60153, USA; 2 Department of Molecular Pharmacology & Therapeutics, Loyola University Chicago, 2160 South First Avenue, Maywood, IL 60153, USA; 3 Hines VA Medical Center, Hines, IL, 60141, USA

## Abstract

**Background:**

The gastrointestinal peptide hormone gastrin is known to regulate various cellular processes including proliferation, migration and metastasis in gastrointestinal (GI) cells. The studies described here were undertaken to elucidate in detail the signaling pathways mediating the migratory responses of amidated gastrin (G17) and to understand the involvement of the serine/threonine kinase Glycogen Synthase Kinase-3 beta (GSK3β) in this.

**Results:**

Our results indicate that incubation of gastric cancer cells overexpressing CCK2 receptor (AGSE cells) with G17 results in a dose and time dependent increase of GSK3β^Ser9 ^phosphorylation, indicative of an inhibition of the kinase. Pretreatment with a pharmacological inhibitor of PI3Kinase pathway (Wortmannin) was unable to antagonize G17-induced GSK3β^Ser9 ^phosphorylation, suggesting that this might involve PI3Kinase-independent pathways. Treatment with G17 was also associated with increased Snail expression, and β-catenin nuclear translocation, both of which are GSK3β downstream targets. Pretreatment with a pharmacological inhibitor of GSK3β (AR-A014418) augmented Snail expression and β-catenin nuclear translocation in the absence of G17, whereas overexpression of a phosphorylation deficient mutant of GSK3β (S9A) abrogated Snail promoter induction. These suggested that G17 modulates Snail and β-catenin pathways via inhibiting GSK3β. In addition, overexpression of GSK3β wild type (WT) or S9A mutant inhibited G17-induced migration and MMP7 promoter induction. G17 studies designed following small interference RNA (siRNA)-mediated knockdown of Snail and β-catenin expression indicated a significant reduction of G-17-induced migration and MMP7 promoter induction following combined knockdown of both proteins.

**Conclusion:**

Our studies indicate that inhibition of GSK3β is necessary to activate G17-induced migratory pathways in gastric cancer cells. Inhibition of GSK3β leads to an induction of Snail expression and β-catenin nuclear translocation, both of which participate to promote G17-induced migration.

## Background

Gastric cancer is the second leading cause of cancer-related deaths worldwide [1], and are often characterized as highly aggressive and unresponsive to therapy [2]. The major risk factor contributing to this disease include *Helicobacter pylori *(*H. pylori*) infection, diet as well as genetic background [3,4]. Interestingly, studies during the past two decades have also demonstrated that the gastrointestinal (GI) peptide hormone gastrin might contribute towards the pathobiology of gastric cancers. In addition to regulating gastric acid secretion, mature gastrin (G-17) and its unprocessed intermediate forms progastrin and glycine extended gastrin (Gly-G) can regulate growth in a variety of cancer cells [5,6]. Results from transgenic mice show that mice overexpressing the amidated form of gastrin have increased proliferation of gastric mucosa [7], which can synergize with *Helicobacter *infection leading to the development of invasive gastric cancer [8]. Prolonged hypergastrinemia increases the relative risk of developing colon cancer [9] and might promote adenoma to carcinoma progression [10]. Recent studies have confirmed gastrin to be an essential cofactor for carcinogenesis of gastric corpus [11]. In addition, significantly high levels of plasma gastrin has been reported in patients with gastric cancer, with high expression of gastrin and its receptor (CCK2R) in gastric cancer cell lines [12]. All these studies indicate an important role of gastrin-and its receptor system in mediating gastric cancer.

Glycogen Synthase Kinase-3 beta (GSK3β) is a ubiquitously expressed serine/threonine kinase, which is active in resting epithelial cells [13]. Phosphorylation of the enzyme on tyrosine residues is required for its activity [14]. Stimulation of cells by agonists leads to an inactivation of GSK3β primarily via phosphorylation of the serine 9 residue [15]. Earlier studies have linked GSK3β in modulating cellular migration [16,17]. In addition, GSK3β is necessary to maintain the epithelial architecture, inhibition of which results in acquisition of a more mesenchymal morphology, termed as epithelial-mesenchymal transition (EMT) [18], a phenomenon necessary for both normal development as well as progression of malignant epithelial tumors [19]. GSK3β can maintain this epithelial morphology via inhibiting the expression of Snail (mediator of EMT) and thus maintaining high E-cadherin expression [20,21]. GSK3β can inhibit Snail expression via inhibiting its transcription [18], as well as regulating Snail degradation and nuclear translocation [22]. Snail has been shown to induce expression of matrix metalloproteinases (MMPs) in cancer cells leading to increased invasion [23]. Snail and its homologue Slug is expressed in gastric cancer, both of which are involved in repression of E-cadherin expression [24,25]. Various other downstream targets of GSK3β have been reported, of which its role in regulating Wnt/β-catenin signaling is well established. In the presence of axin and functionally active Adenomatous Polyposis Coli (APC), GSK3β phosphorylates β-catenin at specific N-terminal residues and targets it toward the ubiquitin-proteasomal degradation pathway. Mutation of either APC or β-catenin itself or activation of signaling pathways that inhibit GSK3β results in stabilization of β-catenin. Once stabilized, β-catenin translocates to the nucleus, and via interaction with transcription factors of the T cell factor/lymphoid enhancer factor (TCF/LEF) family, activates target gene transcription [26]. β-catenin expression has also been detected in the invasive front [27] of the tumors. Several recent studies have demonstrated involvement of GSK3β in mediating different pathways in gastric cancer cells [28,29], and an inhibition of the kinase following *H. pylori *infection [30].

Despite an apparent connection of gastrin in gastric cancer progression, the detailed mechanism by which gastrin mediates its effects is still unclear. In addition to stimulating proliferation, recent studies have shown that G17 as well as its unprocessed forms can activate migration as well as invasion [31-33] which are prerequisites for *in vivo *metastasis. Our earlier studies in gastric cancer cells have demonstrated that G17-induced migration involves an activation of the Mixed-Lineage-Kinase 3/JNK1 signaling axis [31]. Due to a close connection of GSK3β in regulating cell migration, we designed these studies to understand its role in G17-induced migration. Our studies show that incubation with G17 increases GSK3β^Ser9 ^phosphorylation in a transient manner, which was also associated with a corresponding increase in the expression and promoter activation of Snail and an increase in the nuclear translocation of β-catenin. Inhibition of GSK3β via a pharmacological inhibitor resulted in increased Snail expression and β-catenin nuclear translocation in the absence of G17 and overexpression of a phosphorylation deficient mutant of GSK3β (S9A) antagonized G17-mediated induction of Snail promoter. Similarly, ectopic overexpression of Wild type (WT) or S9A mutant of GSK3β antagonized G17-induced migration and MMP7 promoter induction. Our studies also indicate that, combined knockdown of Snail and β-catenin by small interference RNA (siRNA) significantly attenuated G17-induced migration and MMP7 transcription. These studies indicate that G17 modulates Snail and β-catenin pathways via inhibiting GSK3β, both of which in turn participate to mediate G17-induced migration.

## Results

### Effect of Gastrin (G17) on GSK3β^Ser9 ^phosphorylation

**Figure 1:**
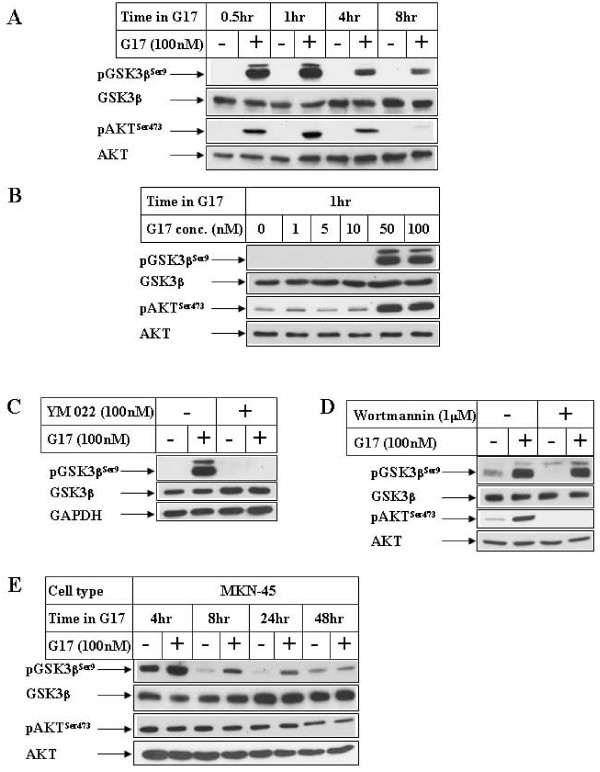
**Effect of G17 on GSK3β^Ser9 ^phosphorylation in gastric cancer cells**. **(A) **Confluent AGSE cells were treated in the absence (-) or presence (+) of 100 nM G17 in serum free media for the indicated periods of time. Equal amounts of total protein were fractionated by SDS-PAGE and subjected to Western Blot analysis utilizing antibodies against phospho-GSK3β^Ser9^, total GSK3β, phospho-AKT^Ser473 ^and total AKT. **(B) **AGSE cells were treated with increasing concentrations of G17 for 1 hour followed by Western Blot analysis with the antibodies indicated. **(C) & (D) **Western Blot analysis of AGSE cells with the indicated antibodies, treated with 100 nM G17 for 1 hour following an overnight pretreatment with 100 nM YM 022 **(C) **or 1 μM Wortmannin **(D)**. **(E) **MKN45 cells treated as in A were harvested at different time points following G17 treatment and analyzed by Western Blots utilizing the antibodies indicated.

In order to determine the role of G17 on GSK3β pathway, Western Blot analysis was performed with G17-treated gastric cancer cells overexpressing the CCK2 receptor (AGSE) [34]. These indicated a time (Fig 1A, pGSK3β^Ser9 ^panel) and dose-dependent (Fig 1B) increase in GSK3β Ser9 phosphorylation, which was maximal after 1 hour of G17 treatment. Pretreatment with an antagonist of the CCK2R (YM 022), inhibited G17-indcued GSK3β^Ser9 ^phosphorylation (Fig 1C), indicating that this is mediated via CCK2R pathway. Since Ser 9 phosphorylation is an inhibitory phosphorylation site of GSK3β, these studies indicated a G17-induced inhibition of GSK3β pathway, possibly via activation of an upstream kinase (for example AKT). In fact, Western analysis also showed an increase in AKT^Ser473 ^phosphorylation (Figs 1A, B pAKT^Ser473 ^panel) corresponding to the time of GSK3β^Ser9 ^phosphorylation, indicating a simultaneous activation of AKT. However, pretreatment of the cells with PI3Kinase inhibitor (Wortmannin) was unable to antagonize G17-induced GSK3β^Ser9 ^phosphorylation (Fig 1D, compare lanes 2 and 4, pGSK3β^Ser9 ^panel), although it completely antagonized AKT phosphorylation (pAKT^Ser473 ^panel). In addition, treatment of another gastric cancer cell line (MKN45) with G17 showed an increase in GSK3β^Ser9 ^phosphorylation without any increase in AKT phosphorylation (Fig 1E). These studies indicated that G17-induced increase in GSK3β^Ser9 ^phosphorylation might involve a PI3 Kinase independent pathway.

### Effect of G17 on Snail expression

**Figure 2:**
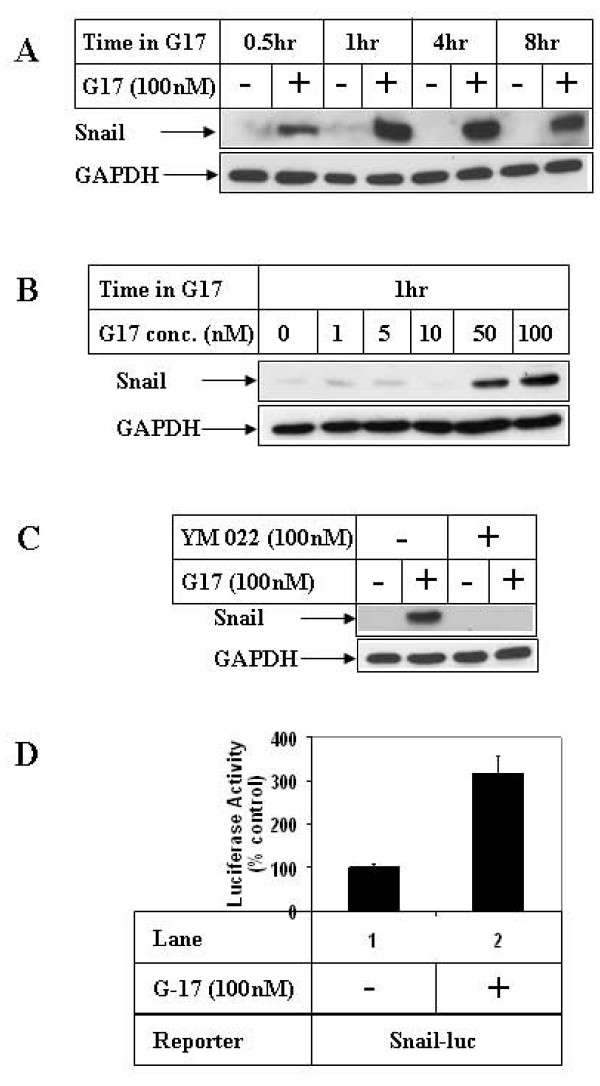
**Effect of G17 on Snail expression in gastric cancer cells**. **(A) **AGSE cells were treated as in 1A or **(B) **1B above and subjected to Western Blot analysis utilizing antibodies against Snail and GAPDH (as control). **(C) **Western Blot analysis of cell extracts with the indicated antibodies, treated with 100 nM G17 for 1 hour, following an overnight pretreatment with 100 nM YM 022. **(D) **Subconfluent AGSE cells were transiently transfected with Snail-luciferase vector (Snail-luc) along with β-Gal vector (for normalization of transfection). Forty-eight hours after transfection, cells were treated overnight in the presence (+) or absence (-) of 100 nM G17, and luciferase and β-Gal assays were performed. The RLU/β-Gal values were represented as percent control, considering the untreated samples as 100%. Each transfection was performed in triplicate, and the data represent the mean ± SD of at least two independent experiments.

To understand the consequences of G17-mediated inhibition of GSK3β, G17 studies were performed to determine changes in the expression of GSK3β-downstream target Snail. Incubation of AGSE cells with G17 resulted in an increase in Snail protein expression in a time (Fig 2A) and dose-dependent (Fig 2B) manner, which was also associated with an increase in Snail transcription (Fig 2D). In addition, G17 induction of Snail expression was mediated via CCK2R, since pretreatment with YM 022 abolished G17-induced Snail expression (Fig 2C).

### G17 induces Snail expression and β-catenin nuclear translocation via inhibiting GSK3β

**Figure 3:**
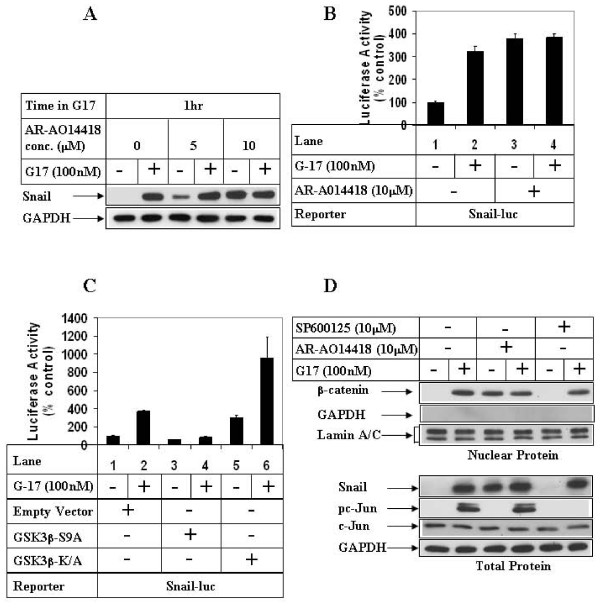
**Effect of GSK3β inhibition on G17-induced Snail expression and β-catenin nuclear translocation**. **(A) **AGSE cells were treated with (+) or without (-) 100 nM G17, following an overnight pretreatment with either none (lanes 1, 2), 5 μM (lanes 3, 4) or 10 μM (lanes 5, 6) AR-A014418. Western Blot analysis was then performed with the antibodies indicated. **(B) **Luciferase (with Snail-luc) and β-Gal assays were performed as in 2D following a 1 hour pretreatment with AR-A014418. **(C) **AGSE cells were co-transfected with Snail-luc and β-Gal vectors along with either Empty vector (lanes 1, 2), GSK3β-S9A mutant vector (lanes 3, 4) or GSK3β-K/A mutant vector (lanes 5, 6). Luciferase and β-Gal assays were performed after G17 treatment as in 2D. Each transfection (3B, 3C) was performed in triplicate, and the data represent the mean ± SD of at least two independent experiments. **(D) Upper Panel**: Confluent AGSE cells were treated with G17 for 8 hours after an overnight pretreatment with none (lanes 1, 2), or AR-A014418 (lanes 3, 4) or SP600125 (lanes 5, 6). At the end of treatment, nuclear protein was isolated and subjected to Western Blot analysis with antibodies against β-catenin, GAPDH (cytoplasmic protein) or Lamin A/C (nuclear protein). **Lower Panel**: Cells were pretreated as in the upper panel, followed by 1 hour G17 treatment and Western Blot analysis.

In order to determine whether G17 increased Snail expression via inhibiting GSK3β, G17 studies were performed following pretreatment of the cells with a pharmacological inhibitor of GSK3β (AR-A014418) [35]. These studies showed an induction of Snail expression following pretreatment with two different concentrations of AR-A014418 (AR) in the absence of G17 (Fig 3A, compare lanes 1, 3 and 5). Pretreatment with 5 μM of AR produced synergistic effects with G17 on inducing Snail expression (compare lanes 3 & 4), whereas at 10 μM AR increased Snail expression to maximal levels without any synergism (compare lanes 5 & 6). Similarly, AR pretreatment by itself increased Snail transcription maximally, without any synergistic effect when combined with G17 (Fig 3B). More mechanistic studies designed following ectopic overexpression of GSK3β showed that overexpression of a phosphorylation-deficient kinase active mutant of GSK3β (S9A) significantly attenuated G17-mediated induction of Snail transcription (Fig 3C, compare lanes 2 and 4). Overexpression of a kinase deficient mutant of GSK3β (K/A), on the other hand increased Snail transcription in the absence of G17 (compare lanes 1 and 5), and produced synergistic effects when treated with G17 (compare lanes 5 and 6). In earlier studies we have demonstrated that G17 treatment increases β-catenin nuclear translocation, without any increase in the expression of total β-catenin protein [36]. Western Blot analysis of nuclear extracts also showed an increase in β-catenin nuclear translocation following AR pretreatment in the absence of G17 (Fig 3D, upper panel, compare lanes 1 and 3), which was equal to the G17-treated levels (lanes 3 & 4). The same extracts were also blotted with GAPDH (cytoplasmic protein) and Lamin A/C (nuclear protein) to show the purity of the nuclear preparation. To understand any crosstalk between MLK3/JNK1 axis [31] and GSK3β axis, Snail and β-catenin studies were performed following pretreatment with the pharmacological inhibitor of JNK (SP600125). These studies indicated a complete inhibition of JNK downstream c-Jun phosphorylation with SP600125 (Fig 3D, lower panel, compare lanes 2 and 6, pc-Jun panel). SP600125 however, was unable to inhibit G17-mediated induction of Snail expression (compare lanes 2 and 6, Snail panel) or β-catenin nuclear translocation (compare lanes 2 and 6, β-catenin panel). These suggested that G17-mediated activation of MLK3/JNK1 and inhibition of GSK3β might be parallel pathways operating independent of each other.

### G17-induced migration involves GSK3β inhibition

**Figure 4:**
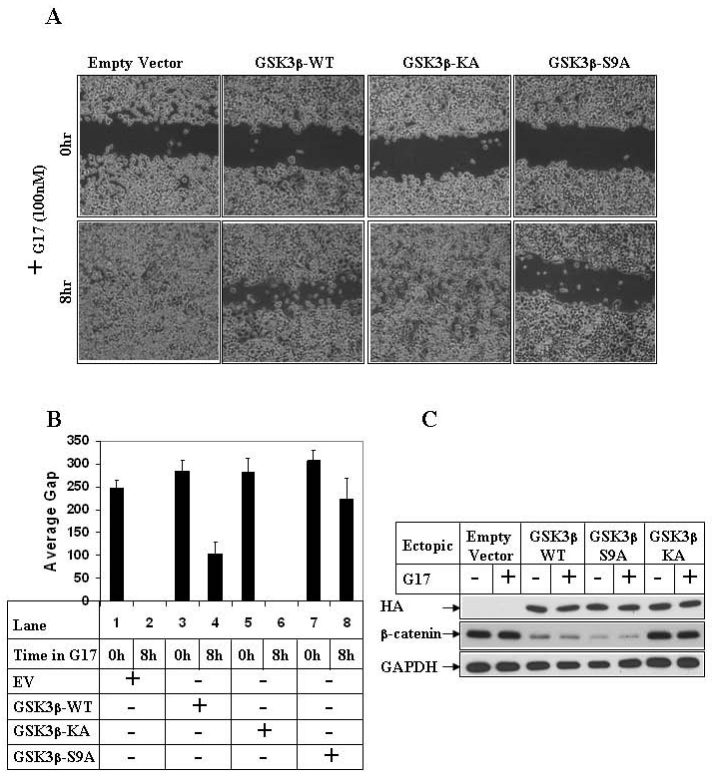
**Effect of overexpression of GSK3β on G17-induced migration**. **(A)**. Subconfluent AGSE cells were transiently transfected with Empty Vector, GSK3β-WT, GSK3β-KA mutant or GSK3β-S9A mutant vectors. The cells were wounded linearly 48 hours post-transfection and, after an overnight recovery following wounding, they were treated with G17 and pictures obtained at the indicated times. **(B) **AGSE cells were transfected as in 4A followed by G17 treatment and wound healing assay. The distance of migration of the wounded edges for each time point were measured at several places and the average distance was represented by bar diagrams as "Average Gap". **(C) **AGSE cells transfected in A and treated with G17 were analyzed for protein expression. Western Blot analysis was performed with an HA.11 antibody to detect ectopic HA-tagged GSK3β proteins and with β-catenin and GAPDH antibodies to detect the corresponding endogenous proteins.

To understand whether G17-induced inhibition of GSK3β was critical to induce migration, wound-healing assays were carried out following overexpression of either wild-type or mutant forms of GSK3β. As shown in Fig 4A, G17-induced migration results in wound closure in the cells overexpressing an empty vector or GSK3β-K/A mutant (8 hr, Empty vector and GSK3β-KA panels). Ectopic overexpression of GSK3β-WT or GSK3β-S9A on the contrary, significantly inhibited G17-induced migration (compare GSK3β-WT, S9A and Empty vector panels). The average gap of migration in these cells were also measured and plotted as graphs, which indicated a complete wound closure at 8 hrs of G17 treatment with Empty vector (Fig 4B, lane 2) and GSK3β-K/A (lane 6), and an inhibition of migration with GSK3β-WT (lane 4) and GSK3β-S9A (lane 8). Western Blot analysis of these cell extracts is shown in Fig 4C, which indicates the expression of the various ectopic GSK3β forms. In these samples, overexpression of GSK3β-WT and S9A resulted in a decrease in the expression of endogenous β-catenin (β-catenin panel, lanes 3-6), suggesting that these ectopic proteins retain GSK3β activity.

### G17-induced migration involves Snail and β-catenin pathways

**Figure 5:**
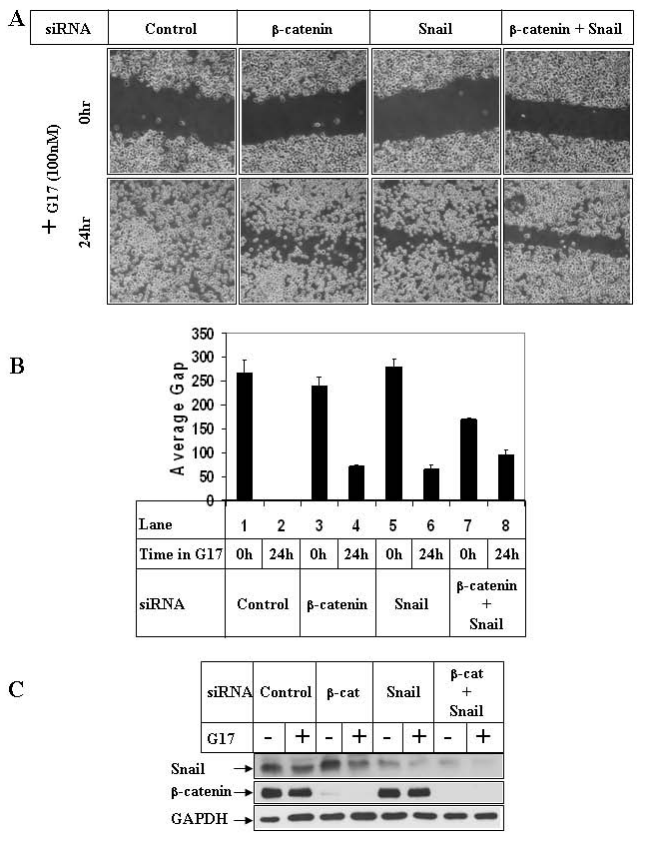
**Effect of knockdown of β-catenin and Snail expression on G17-induced migration**. **(A)**. Subconfluent AGSE cells were transiently transfected with 100 nM each of either control-siRNA, or β-catenin-siRNA, or Snail-siRNA or a combination of β-catenin and Snail siRNA. They were wounded 48 hours post-transfection and treated with G17 and pictures were obtained as described under 4A. **(B) **The distance of migration of the wounded edges were measured as in 4B and represented as "Average Gap". **(C) **AGSE cells transfected in A and treated with G17 were analyzed by Western Blot analysis utilizing the indicated antibodies.

Since GSK3β inhibition in these cells was enough to induce Snail expression and β-catenin nuclear translocation (Figs 3A, D), and inhibition of GSK3β was necessary for G17-induced migration (Fig 4A), it was conceivable that Snail and β-catenin are involved in G17-induced migration. To address this possibility, wound-healing assays were performed following knockdown of endogenous Snail and β-catenin protein expression (alone or in combination) utilizing corresponding siRNAs. Transfections of Snail or β-catenin siRNA lead to a significant decrease in the expression of the respective proteins as shown in Fig 5C. Wound-healing assays performed under these conditions indicated a complete closure of the wound following G17 stimulation in the presence of control siRNA (Fig 5A, control siRNA panel), which was inhibited significantly following combined knockdown of Snail and β-catenin (β-catenin + Snail panel). Knockdown of either protein alone produced only partial effects. The average gap of migration is indicated in Fig 5B, which also shows a complete closure with control siRNA (lane 2), partial inhibition with Snail or β-catenin siRNA alone (lanes 4, 6) and significant inhibition with the combination (lane 8).

### G17-mediated induction of MMP7-promoter activation involves GSK3β, Snail and β-catenin

**Figure 6:**
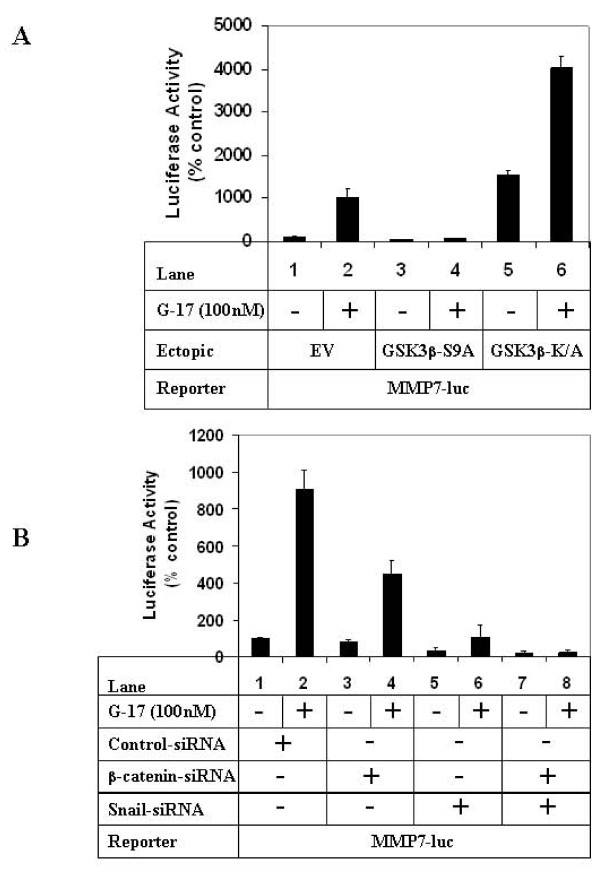
**Effect of modulation of GSK3β, β-catenin and Snail pathways on G17-induced MMP7 transcription**. **(A)**. Subconfluent AGSE cells were co-transfected with MMP7-luciferase and β-Gal vectors along with either EV (lanes 1, 2), GSK3β-S9A (lanes 3, 4) or GSK3β-K/A (lanes 5, 6) mutants. Luciferase and β-Gal assays were performed after G17 treatment as described under 2D. **(B) **AGSE cells were transfected as in A along with either control-siRNA (lanes 1, 2), β-catenin-siRNA (lanes 3, 4), Snail-siRNA (lanes 5, 6) or a combination of β-catenin and Snail-siRNA (lanes 7, 8). G17 treatment was performed as described under 2D, followed by luciferase and β-Gal assays. Each transfection (A and B) was performed in triplicate, and the data represent the mean ± SD of at least two independent experiments.

To elucidate further the mechanism by which G17/GSK3β axis mediated migration, studies were also designed with the MMP7 promoter which was shown to be induced by G17 in our earlier studies [31]. Treatment with G17 resulted in an increase in MMP7 promoter activity when transfected with an empty vector (Fig 6A, compare lanes 1, 2), which was inhibited significantly in the presence of GSK3β-S9A (compare lanes 2, 4). Overexpression of GSK3β-K/A on the other hand, increased MMP7-promoter activity in the absence of G17 (lane 5), and produced synergistic effects when combined with G17 (lane 6). These suggested the involvement of GSK3β in mediating G17-induced MMP7 promoter induction. Since Snail and β-catenin are the two downstream targets of GSK3β mediating G17-induced migration (Fig 5A), it is likely that they are also involved in inducing MMP7 promoter activity. Luciferase assays indicated a partial inhibition of G17-induced MMP7 promoter activity following knockdown of β-catenin expression alone (Fig 6B, compare lanes 2 and 4), which was significantly inhibited following knockdown of Snail alone (lane 6) or with combined knockdown of Snail and β-catenin (lane 8).

## Discussion

The GI peptide hormone gastrin (G17 and its unprocessed forms) can regulate various cellular processes involved in cancer [37,38]. The studies described here were designed to elucidate in depth the mechanism by which G17 induces migration in gastric cancer cells, which in our earlier studies have shown to involve an activation of the MLK3 and JNK1 signaling axis [31]. In this axis G17-induced activation of JNK1 leads to phosphorylation and activation of c-Jun, leading to induction of MMP7 transcription and increased migration. The current studies were performed to determine whether the serine/threonine kinase GSK3β plays any role in this, due to its close connection in regulating cellular migration. A crosstalk of MLK3 pathway with GSK3β has also been reported earlier [39]. GSK3β has been shown to regulate migration both in a positive and negative manner. For example inactivation of GSK3β can increase migration in fibroblasts [16], and induce EMT in nontumorigenic breast epithelial cells [18]. In other studies, GSK3β was shown to promote cancer cell migration by cooperating with h-prune [40], or with small GTPase Rac [41]. To obtain a mechanistic insight towards the role of GSK3β in G17-induced migration, overexpression studies were performed with either wild-type or mutant forms of the kinase. As shown in Fig 4A, ectopic overexpression of GSK3β-WT as well as S9A mutant significantly attenuated G17-induced migration. MMP7 is known to mediate migration of gastric cancer cells [42], the transcription of which was induced by G17 [31]. Studies described here also revealed an inhibition of G17-induced MMP7 promoter activity following overexpression of GSK3β-S9A (Fig 6A), which was increased following overexpression of GSK3β-KA in the absence of G17.

In many cells, GSK3β is constitutively active, which can be inactivated by various signaling mechanisms including Wnt signaling pathway [26,43] and PI3K/AKT pathway [44]. Although the detailed mechanism how Wnt pathway inactivates GSK3β is still unclear, PI3K/AKT inhibits GSK3β via increasing its Ser 9 phosphorylation [45]. In our studies, treatment with G17 also produced an increase in GSK3β^Ser9 ^phosphorylation (Figs 1A, 1B), suggesting an inactivation of the kinase during G17/CCK2R activation. This was associated with a corresponding increase in AKT^Ser473 ^phosphorylation, indicating the possibility that G17 might induce GSK3β^Ser9 ^phosphorylation and downstream cellular responses via PI3K/AKT activation. However, pretreatment with Wortmannin (pharmacological inhibitor of PI3Kinase), was unable to antagonize G17-induced GSK3β^Ser9 ^phosphorylation (Fig 1D, pGSK3β^Ser9 ^panel), despite a complete inhibition of AKT^Ser473 ^phosphorylation (pAKT^Ser473 ^panel). In addition, treatment of another gastric cancer cell line (MKN45) with G17 produced an increase in GSK3β^Ser9 ^phosphorylation without any effect on AKT phosphorylation (Fig 1E). These results suggested that G17-induced increase of GSK3β^Ser9 ^phosphorylation was mediated via PI3K/AKT independent pathway. AKT-independent phosphorylation of GSK3β has been reported earlier [29,46], including those mediated by members of the PKC pathway [47]. It will thus be important to determine the contribution of any of these signaling pathways in mediating G17-induced GSK3β^Ser9 ^phosphorylation.

The detailed mechanism by which GSK3β regulates migration is still unknown and might involve specific downstream targets. Since Snail and β-catenin are both downstream targets of GSK3β [22,26], which are also involved in mediating EMT, migration and proliferative responses, the next set of studies were specifically focused on understanding the role of these molecules on G17-induced events. Snail has been shown to mediate inflammation-linked migration in cancer cells [48] and promote EMT, a phenomenon that is a prerequisite for cellular migration, invasion and normal development process [19,49]. Our studies with G17 indicated a transient increase in Snail protein expression as well as transcription (Figs 2A, 2D) corresponding to the time of increased GSK3β^Ser9 ^phosphorylation. These studies also show that the increase in Snail expression was mediated via inhibition of GSK3β pathway, since pretreatment with a pharmacological inhibitor of GSK3β (AR-A014418) induced Snail expression and transcription in the absence of G17 (Figs 3A, B). In addition, ectopic overexpression of GSK3β-S9A inhibited G17-induced Snail transcription (Fig 3C). The other GSK3β target β-catenin is considered to be a major oncoprotein and a mediator of conventional Wnt/β-catenin pathway [26]. In normal cells, constitutively active GSK3β negatively regulates Wnt/β-catenin signaling via phosphorylation-induced degradation of β-catenin, thus limiting β-catenin expression and activation of Wnt/β-catenin signaling. Interestingly, in our studies, despite an induction of GSK3β^Ser9 ^phosphorylation (indicating inactivation), we observed no increase in β-catenin total protein expression at any time point following G17 treatment as reported earlier [36]. However, Western analysis with nuclear extracts showed a distinct increase in β-catenin nuclear translocation with G17, which was also induced following pretreatment with AR in the absence of G17 upto similar levels (Fig 3D, upper panel). This indicated a link between GSK3β inactivation and β-catenin nuclear translocation. The results from these studies and those of others thus suggest that GSK3β activation can regulate β-catenin signaling at two distinct levels: (i) it inhibits β-catenin expression via activating the conventional degradation pathway (ii) it inhibits β-catenin nuclear translocation via a yet unknown mechanism. The former event seems to be lacking in these cells with G17 stimulation, since G17 does not lead to an increase in β-catenin total protein expression [36]. The second event is present in the G17 pathway, since inhibition of GSK3β by AR increases β-catenin nuclear translocation. The mechanism how GSK3β inhibits β-catenin nuclear translocation is still unclear, and might involve a similar phosphorylation dependent mechanism as was reported in the nuclear export of cyclin D1, another GSK3β downstream target [50]. Recent studies by another group have demonstrated that p21-activated kinase 1 (PAK1) is also involved in regulating various steps of β-catenin signaling and migration following G17 stimulation [51]. It is thus tempting to speculate that a crosstalk between GSK3β and PAK1 might be mediating this process. Since JNK pathway can regulate β-catenin nuclear translocation [52] and G17 can activate JNK [31], G17 studies were also performed following pretreatment with an inhibitor of JNK pathway (SP600125). SP600125, however, was unable to show any increase in β-catenin nuclear translocation or Snail induction (Fig 3D) despite a complete antagonism of c-Jun phosphorylation, suggesting that these two proteins are specifically regulated by GSK3β axis.

**Figure 7:**
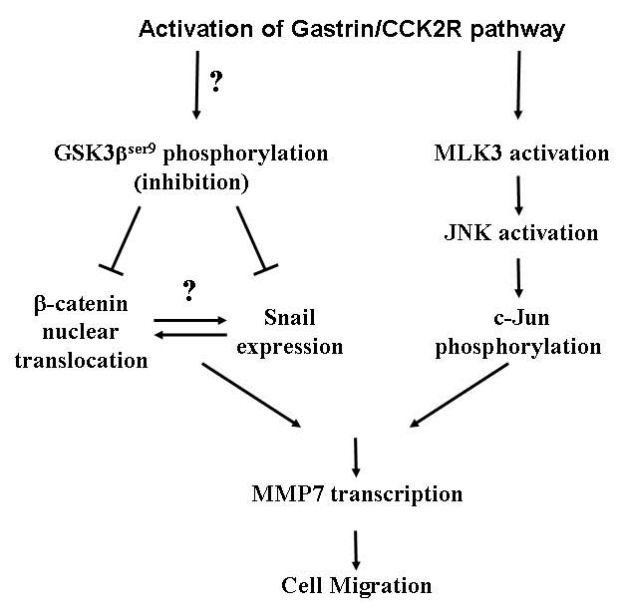
**Model representing the signaling pathway of G17-induced migration**. Stimulation of G17/CCK2R pathway leads to activation of two separate signaling axes: (i) an activation of MLK3/JNK1 axis, which via activation of its downstream transcription factor c-Jun induces MMP7 transcription leading to increased migration; (ii) an induction of GSK3β^Ser9 ^phosphorylation (inhibition of axis) via a PI3Kinase-independent (and yet unknown) mechanism. This leads to an increase in Snail protein expression and β-catenin nuclear translocation, combination of which lead to increased MMP7 transcription and cell migration.

To understand whether Snail and β-catenin were specific downstream targets of GSK3β to mediate G17-induced migration, G17 studies were performed following siRNA-mediated knockdown of endogenous Snail or β-catenin expression. Knockdown of either protein alone produced a partial inhibition of G17-induced migration (Fig 5A) as well as MMP7 promoter induction (Fig 6B). However, combined knockdown of both proteins significantly antagonized these events. Although a crosstalk between β-catenin and Snail has been reported earlier [53], we were unable to detect any endogenous interaction between these two proteins (data not shown). Further studies are needed to elucidate how Snail and β-catenin coordinate with each other to mediate G17 effects. Interestingly, our earlier studies have indicated the involvement of MLK3/JNK1/c-Jun axis in regulating MMP7 promoter activation and migration following G17 stimulation [31]. Taken together it seems that G17 stimulation operates via two independent signaling axes which ultimately leads to migration: one involves activation of MLK3/JNK1 axis which operates via activation of c-Jun, and the second one involves inhibition of GSK3β axis leading to an induction of Snail expression and β-catenin nuclear translocation. Both of these axes converge to induce MMP7 transcription and migration (Fig 7), and thus represent two potential targets for future drug development.

## Conclusions

The present study demonstrates that G17-induced migration and MMP7 promoter induction in gastric cancer cells involve an inhibition of GSK3β pathway. G17-induced inhibition of GSK3β leads to an increase in Snail expression and β-catenin nuclear translocation, both of which collectively mediate the migratory response. Thus β-catenin and Snail serve as two downstream targets of GSK3β in G17-induced migration pathway. However, this G17/GSK3β/Snail-β-catenin axis seems to be independent of a previously identified G17/MLK3/JNK1 axis [31] and both operate parallel to each other to induce migration. Targeting of both of theses axes might be beneficial to antagonize G17-induced migratory effects in gastric cancer.

## Methods

### Reagents

DMEM, LipofectAMINE 2000 and β-Galactosidase assay kit were purchased from Invitrogen (Carlsbad, CA); amidated gastrin (G17) from Bachem (King of Prussia, PA) and the luciferase assay kit from Promega (Madison, WI). The antibodies utilized were obtained from the following sources: GSK3β, pGSK3β^Ser9^, AKT, pAKT^Ser473^, pc-Jun, c-Jun, Snail and Lamin A/C (Cell Signaling Technology, Danvers, MA), GAPDH (Ambion, Austin, TX), β-catenin (BD Biosciences, San Jose, CA), HA.11 (Covance, Berkeley, CA). The CCK2R antagonist YM 022 was from Tocris Bioscience (Ellisville, MO), PI3 Kinase inhibitor Wortmannin and GSK3β inhibitor AR-A014418 from EMD Biosciences (Gibbstown, NJ) and JNK inhibitor SP600125 from Alexis Biochemicals, Axxora (San Diego, CA). The AGSE cells were obtained from Dr. Timothy C. Wang (Columbia University Medical Center, New York, NY) as described earlier [34,36]. The MMP7-luciferase promoter construct was obtained from Dr. Howard Crawford (Stony Brook University, Stony Brook, NY) [54] and the Snail-luciferase promoter was obtained from Dr. Antonio Garcia de Herreros (Universitat Pompeu Fabra, Barcelona, Spain) [18]. The HA-tagged GSK3β expression vectors (WT, K/A and S9A) were from Dr. James R Woodgett (University of Toronto, Toronto, Canada) [55].

### Cell Culture

The AGSE cells used in these studies were derived from AGS cells stably overexpressing CCK2R as described previously [34]. These cells were maintained in DMEM supplemented with 10% FBS and 100 IU/ml penicillin as reported [31,36]. Wherever indicated, confluent populations of cells were treated with 100 nM G17 in serum deficient media, and subjected to Western Blot analysis, luciferase or migration assays. In the studies with various inhibitors, cells were pretreated with the specific inhibitors followed by G17 treatment.

### Luciferase assays

Subconfluent populations of cells were transiently transfected with Snail-luciferase [18] or MMP7-luciferase reporter constructs [54] along with β-Galactosidase vector (to correct for transfection efficiency) using lipofectAMINE 2000 as per manufacturer's instructions. To determine the effect of overexpression of GSK3β (WT or mutants) or knockdown of Snail and β-catenin, the corresponding overexpression vectors or siRNAs respectively were co-transfected along with the luciferase reporters. Each transfection was performed in triplicate and each experiment was repeated at least twice. Following 48 hours of recovery in the growth medium, the transfected cells were treated with either vehicle or G17 for 24 hours. Luciferase and β-Galactosidase (β-Gal) assays were performed as described [31] using a luminometer (Berthold Technologies, Centro XS^3 ^LB 960) and a plate reader (Power Wave XS, Biotek) respectively. The results obtained were calculated as the ratio of relative light units (RLU) to β-Gal values (RLU/β-Gal) and expressed as % increase compared to controls.

### Western Blot analysis

Whole cell extracts were prepared from cells treated with G17 by RIPA extraction buffer, and equal amounts of total protein were subjected to Western Blot analysis utilizing procedures described previously [31,36]. Nuclear protein extraction was performed following protocols as described [56]. Briefly, cells were incubated with a lysis buffer (containing 1% Triton X-100, 50 mM Hepes pH 7.6, 150 mM NaCl, 100 mM NaF, 50 mM Na-pyrophosphate, 4 mM EDTA and 10 mM Na_3_VO_4, _supplemented with protease inhibitors) and rotated at 4°C for 30 minutes. This was followed by centrifugation at 13,000 rpm for 15 minutes to pellet nuclei, washing the nuclear pellet once with wash buffer (containing lysis buffer + 25% glycerol) and centrifugation again. To obtain nuclear extract, the nuclear pellet was lysed in nuclear lysis buffer (wash buffer + 300 mM NaCl), sonicated and incubated on ice for 30 minutes followed by centrifugation. Western Blot analysis was performed with equal amounts of nuclear protein with the indicated antibodies. To determine the purity of the nuclear preparation, they were blotted with antibodies against GAPDH (cytoplasmic protein) and Lamin A/C (nuclear protein).

### Small interference RNA (siRNA)

The β-catenin siRNA [57] was synthesized from Dharmacon (Lafayette, CO) and the Snail siRNA was from Invitrogen (Carlsbad, CA) [58]. The control-siRNA was from Ambion (Austin, TX). siRNA transfection was performed using lipofectAMINE 2000 as per manufacturer's instructions following protocols described earlier [57]. G17 treatment was performed after 48 hours of siRNA transfection, and the cells were then subjected to Western Blot, luciferase or migration assays.

### Wound healing assay

These were performed following procedures described earlier [31]. Confluent populations of cells were wounded linearly using a small pipette tip, washed with PBS and treated with the various agents in serum deficient media for various periods of time. For overexpression experiments, cells were transfected with the corresponding overexpression vectors or siRNAs, wounded 48 hrs following transfection, and allowed to recover overnight before adding G17. The migratory cells were visualized and photographed using inverted phase-contrast microscopy (Axiovert 200 inverted microscope, Zeiss), interfaced with a camera (Axiocam) and the image analyzer software (Axiovision, Zeiss). To estimate relative migration, the unclosed distances at 3 points in each scratch were measured using the Axiovision software, their average calculated and plotted as "Average Gap".

## Abbreviations

APC: Adenomatous Polyposis Coli; β-Gal: β-Galactosidase; CCK2R: CCK2 receptor; EV: Empty Vector; EMT: epithelial-to-mesenchymal transition; G-17: amidated gastrin; G-Gly: glycine-extended gastrin; GI: Gastrointestinal; GSK3β: Glycogen Synthase Kinase 3beta; *H. pylori*: *Helicobacter pylori*; JNK: c-Jun NH2-terminal Kinase; MLK3: Mixed lineage kinase-3; MMPs: matrix metalloproteinases; PAK1: p21-activated kinase 1; PBS: Phosphate buffer saline; PI3Kinase: phosphatidylinositol-3 kinase; RLU: relative light units; siRNA: small interference RNA; TCF/LEF: T cell factor/lymphoid enhancer factor; WT: Wild Type;

## Competing interests

The authors declare that they have no competing interests.

## Authors' contributions

PM performed Western blot analysis, Luciferase analysis, overexpression and migration experiments. SS performed some of the gastrin-related migration and Western experiments in various gastrointestinal cells, obtained photographs of migration and contributed to the editing of the manuscript. AR helped with the analysis of the GSK3β overexpression experiments and provided intellectual input in this collaborative study. BR contributed to the overall study design, interpretation of the results during all phases and drafted/edited the final manuscript. All authors read and approved the final manuscript.
